# Editorial: The diversity of the plasticitome

**DOI:** 10.3389/fnsyn.2026.1969861

**Published:** 2026-09-10

**Authors:** Christina You Chien Chou, Serena M. Dudek, Clive R. Bramham, Hyungju Park, P. Jesper Sjöström

**Affiliations:** 1 Centre for Research in Neuroscience, Brain Repair and Integrative Neuroscience Program, Department of Medicine, Department of Neurology and Neurosurgery, The Research Institute of the McGill University Health Centre, Montreal General Hospital, Montreal, QC, Canada; 2 National Institute of Environmental Health Sciences, National Institutes of Health, Durham, NC, United States; 3 Department of Biomedicine and Mohn Research Center for the Brain, University of Bergen, Bergen, Norway; 4 Korea Brain Research Institute (KBRI), Daegu, Republic of Korea

**Keywords:** diversity, learning, memory, neuron specificity, plasticitome, plasticity, synapse specificity

## Introduction

Synaptic plasticity is widely regarded as a key cellular substrate for learning, memory, and adaptive circuit function. For decades, research has centered on a handful of canonical excitatory connections, such as Schaffer collateral-CA1 synapses (Bliss and Collingridge, 1993), parallel fiber-cerebellar Purkinje cell synapses (Ito, 1989), and unitary neocortical pyramidal neuron connections (Markram et al., 1997). These model synapses have provided profound mechanistic insights, but growing evidence from a broader range of synapses, cell types, and circuits indicates that the induction, expression, and function of synaptic plasticity depend critically on synapse type, neuronal identity, circuit architecture, neuromodulatory state, developmental stage, and pathological context (Markram et al., 1998; Reyes et al., 1998; Toth and McBain, 2000; Blackman et al., 2013; Larsen and Sjöström, 2015). Rather than being governed by a single universal learning rule, synaptic plasticity comprises a rich repertoire of mechanisms that differ across synapses and circuits and may be specialized for their computational demands (Malenka and Bear, 2004).

Building on evidence for synapse-type-specific plasticity, the concept of the plasticitome, analogous to the genome, proteome, and connectome, was introduced as a framework for systematically identifying, classifying, and ultimately understanding the diversity of synaptic learning rules across the brain (Sjöström, 2021; McFarlan et al., 2023). In this view, the plasticitome encompasses the full repertoire of synaptic learning rules together with the biological contexts in which they are engaged, including pre- and post-synaptic cell identity, induction protocol, neuromodulatory state, developmental stage, and disease.

Systematically charting the plasticitome remains challenging because traditional approaches are inherently low throughput, motivating the development of higher-throughput experimental and computational approaches for exploring plasticity across synapse types and circuits (Perin et al., 2011; Emiliani et al., 2015; Annecchino et al., 2017; Field et al., 2020; Campagnola et al., 2022; Kim et al., 2023; Chou et al., 2025). The contributions collected in this Research Topic provide complementary glimpses of this diversity, spanning molecular mechanisms, distinct synapse and cell types, and plasticity across physiological and pathological states.

## Molecular diversity across synapse types

The first group of studies explores how molecular mechanisms generate specialized forms of synaptic plasticity across synapse types and circuits.

Acharya et al. compared the presynaptic active zones of alarm odor-detecting Or56a and food odor-detecting Or22a olfactory sensory neurons in Drosophila. Or56a neurons contained fewer active zones but expressed more Unc13A, consistent with increased presynaptic release, whereas Or22a neurons exhibited a molecular signature associated with lower presynaptic release. Consistent with previous evidence for cell-type-specific homeostatic plasticity in olfactory sensory neurons (Halty-deLeon et al., 2024), only Or22a neurons compensated for reduced presynaptic release by increasing active zone number and Unc13A expression, highlighting distinct molecular strategies that underlie synapse-type-specific plasticity.

Bartol et al. showed that computational modeling can reveal molecular mechanisms of synaptic plasticity that are difficult to probe experimentally. The group used a spatially realistic computational model of hippocampal dendritic spines to investigate what prolongs the lifetime of autophosphorylated Ca^2+^/calmodulin-dependent protein kinase II (CaMKII) following LTP induction. Their simulations showed that CaM-trapping prolongs the lifetime of autophosphorylated CaMKII by hindering dephosphorylation rather than by promoting further autophosphorylation.

Noriega-Prieto et al. showed that insulin-like growth factor I (IGF-I) exerts bidirectional control over NMDA receptor-dependent synaptic plasticity in mouse barrel cortex. Whereas, higher IGF-I concentrations facilitated Hebbian LTP by inducing long-term depression of inhibitory transmission, lower concentrations prevented LTP by inducing long-term depression of excitatory transmission. Together with previous evidence that neuronal activity promotes IGF-I entry into the brain (Nishijima et al., 2010), these findings suggest that physiological fluctuations in local IGF-I levels may dynamically tune cortical plasticity.

Beyond the sensory periphery, odor-guided behaviors also depend on synaptic plasticity in central olfactory circuits. In the mouse olfactory tubercle (OT), reward and punishment induce domain-specific plasticity in the anteromedial (amOT) and lateral (lOT) domains (Sha et al., 2023). Podder et al. showed that although both regions expressed LTP, the neuropeptide orexin selectively lowered the LTP threshold in the amOT, where orexin receptor expression was higher. These findings demonstrate how regional differences in neuromodulation tune a shared plasticity mechanism, potentially supporting distinct behavioral functions.

## Plasticity across cell types and synapses

The second group of studies explores how synaptic learning rules differ across neuronal cell types and synaptic connections.

Chou et al. showed that postsynaptic spiking determines anti-Hebbian long-term depression (LTD) at excitatory synapses onto visual-cortex basket cells, extending previous observations at hippocampal pyramidal-to-basket-cell synapses (Lamsa et al., 2007; Camiré and Topolnik, 2014). Synchronous pre-before-post pairing elicited supralinear dendritic Ca^2+^ signals and LTD, whereas asynchronous pairing did not. High-throughput 2-photon optomapping further revealed opposing plasticity at neighboring synapse types: the same synchronous pairing protocol potentiated pyramidal-to-pyramidal connections but depressed pyramidal-to-basket-cell inputs.

Guinet et al. investigated inhibitory plasticity at GABAergic synapses onto burst-spiking and regular-spiking pyramidal neurons in the subiculum. Whereas associative stimulation induced inhibitory long-term potentiation (iLTP) in both cell types, non-associative stimulation elicited iLTP only in regular-spiking neurons. These findings show that inhibitory plasticity rules depend on postsynaptic cell identity, even among closely related principal neurons.

McFarlan et al. showed that short-term plasticity (STP) of vasoactive intestinal peptide (VIP) interneurons is strongly synapse-type specific in mouse motor cortex. VIP outputs onto Martinotti and basket cells consistently exhibited short-term depression, whereas excitatory inputs to VIP interneurons displayed highly heterogeneous dynamics linked to presynaptic release probability and postsynaptic cell identity. Together with recent work demonstrating that heterogeneous STP provides a substrate for temporally structured population dynamics (Bender et al., 2026), STP diversity emerges as a key substrate of circuit computation.

Severin and Kirkwood discuss how excitatory recruitment of parvalbumin-positive (PV) interneurons regulates inhibitory tone across development, aging, and disease. Synthesizing evidence from the visual cortex and medial temporal lobe, they identify the activity-dependent synaptic protein NPTX2 as a key regulator of PV interneuron excitation. The authors propose that restoring excitatory drive of PV interneurons may provide a therapeutic strategy for limiting network hyperexcitability in neurodegenerative disease.

Nasrallah et al. identified a previously unrecognized form of heterosynaptic N-methyl-D-aspartate receptor (NMDAR) plasticity in dentate granule cells. Burst-timing-dependent potentiation at medial perforant path synapses induced NMDAR LTP not only homosynaptically but also at unstimulated lateral perforant path inputs, where α-amino-3-hydroxy-5-methyl-4-isoxazolepropionic acid receptor (AMPAR)-mediated transmission instead underwent heterosynaptic LTD. These dissociable forms of NMDAR and AMPAR plasticity reveal distinct learning rules for convergent entorhinal inputs onto the same neuron.

## Plasticity across physiological and pathological states

The final group of studies explores how synaptic learning rules are reshaped by development, physiological state, and disease.

Although the mechanisms underlying hippocampal LTP have long been debated (Bliss et al., 2025), Gall et al. argue that many longstanding controversies surrounding hippocampal LTP arise because it is not a single, uniform phenomenon but instead comprises multiple forms that differ across hippocampal regions, sexes, and patterns of activity. Their synthesis shows both the how and the why of hippocampal LTP depend on biological context, illustrating the diversity of the plasticitome.

Feighan et al. reviewed the convergence and divergence of molecular mechanisms underlying Hebbian and homeostatic plasticity, focusing on regulation of AMPARs. By comparing synaptic scaffolding proteins, intracellular signaling pathways, cell-adhesion molecules, and secreted factors, they identify both shared and distinct molecular pathways that shape different forms of synaptic plasticity.

García-Magro et al. showed that diabetes impairs synaptic plasticity in the primary somatosensory cortex by converting whisker stimulation-induced LTP into NMDAR- and metabotropic glutamate receptor-dependent LTD. Local application of IGF-I partially restored LTP, linking impaired IGF-I signaling to altered cortical plasticity and illustrating how metabolic disease can reshape synaptic learning rules.

Halim et al. investigated how pre-sensory spontaneous activity shapes maturation of auditory ribbon synapses before hearing onset. Using pharmacological, genetic, and optogenetic manipulations, they showed that the structure of developing inner hair cell ribbon synapses is bidirectionally regulated by the temporal pattern of activity. Spontaneous activity patterns thus instruct structural plasticity during synaptic maturation.

## Concluding remarks

The studies in this Research Topic highlight the diversity of synaptic plasticity across molecules, synapse types, cell classes, circuits, and physiological and pathological states. Together, they suggest that the general principles governing learning cannot be inferred from any single model system but instead emerge by considering the diversity of synaptic learning rules operating throughout the brain. Rather than being governed by a universal mechanism, synaptic plasticity comprises a rich repertoire of learning rules specialized for the computational demands of individual synapses and circuits.

A major challenge for the future will be to move beyond studying isolated forms of plasticity toward systematically charting the plasticitome: the landscape of synaptic learning rules across brain regions, synapse types, developmental stages, behavioral states, and disease. An initial plasticitome atlas may therefore emerge by systematically combining standardized measurements across synapse types, brain regions, and physiological states into a common experimental framework, for example building on recent large-scale efforts to systematically quantify connectivity and short-term plasticity across neuronal cell types (Campagnola et al., 2022; Chou et al., 2025).

Just as genomics, proteomics, and connectomics have provided unifying frameworks for understanding genes, proteins, and neuronal connectivity, respectively, a comprehensive plasticitome promises a comparable framework for understanding how neural circuits develop, adapt, learn, and malfunction. We hope this Research Topic provides a foundation for increasingly systematic efforts to chart the plasticitome.
